# Clinical Trajectories and Causes of Death in Septic Patients with a Low APACHE II Score

**DOI:** 10.3390/jcm8071064

**Published:** 2019-07-20

**Authors:** Chun-Ta Huang, Sheng-Yuan Ruan, Yi-Ju Tsai, Shih-Chi Ku, Chong-Jen Yu

**Affiliations:** 1 Department of Internal Medicine, National Taiwan University Hospital, Taipei 100, Taiwan; 2 Graduate Institute of Clinical Medicine, National Taiwan University, Taipei 100, Taiwan; 3 Graduate Institute of Biomedical and Pharmaceutical Science, College of Medicine, Fu Jen Catholic University, New Taipei City 242, Taiwan

**Keywords:** acute physiology and chronic health evaluation, intensive care unit, outcome, risk factor, sepsis

## Abstract

Clinical course and mortality in septic patients with low disease severity remain poorly understood and is worth further investigation. We enrolled septic patients admitted to intensive care units (ICUs) between 2010 and 2014 with Acute Physiology and Chronic Health Evaluation II (APACHE II) scores of ≤15. We sought to determine their clinical trajectories and causes of death, and to analyze risk factors associated with in-hospital mortality. A total of 352 patients were included, of whom 89 (25%) did not survive to hospital discharge, at a rate higher than predicted (<21%) by the APACHE II score. Approximately one third (31/89) of non-survivors succumbed to index sepsis; however, more patients (34/89) died of subsequent sepsis. New-onset ICU sepsis developed in 99 (28%) patients and was an independent risk factor for mortality. In addition, septic patients with comorbid malignancy or index infection acquired in the hospital settings were more likely to have in-hospital mortality than those without. In conclusion, septic patients with low APACHE II scores were at a higher mortality risk than expected, and subsequent sepsis rather than index sepsis was the primary cause of death. This study provides insight into unexpected clinical trajectories and outcomes of septic patients with low disease severity at ICU admission and highlights the need for more research and clinical attention in this patient population.

## 1. Introduction

Sepsis is a clinical syndrome caused by a dysregulated host response to infection and manifests as physiologic, biologic, and biochemical abnormalities [1,2]. The definition of sepsis has evolved rapidly since the early 1990s and there have been three major consensus conferences convened to refine its definition [2,3,4]. No matter what the definition of sepsis is, it is a major cause of morbidity and mortality worldwide [5,6,7], and its reported incidence is increasing [5,8,9], likely reflecting an aging population with more comorbidities, and greater awareness and recognition [10,11]. Unanimously, by updating the definition of sepsis we aim to offer consistency for epidemiologic studies and clinical trials, to facilitate earlier recognition and more timely management of patients with sepsis, and to guide an optimal allocation of healthcare resources and proper disposition of patients to intensive care units (ICUs) [2]. After all, the ultimate goal of all the efforts is to improve the outcome of patients with sepsis.

An ICU, specially staffed and equipped, enables monitoring of vulnerable, critically ill patients, provides prompt and proper responses to life-threatening conditions, and ensures efficient support for vital functions. Thus, oftentimes patients with sepsis mandate admission to the ICU to reduce preventable death and disability associated with the sepsis catastrophe. At the time of ICU admission, the Acute Physiology and Chronic Health Evaluation II (APACHE II) scoring system is commonly used to assess disease severity and predict clinical outcome in critically ill patients [12,13,14,15,16]. Clinical vigilance and research interests are usually attracted by septic patients with high APACHE II scores; however, to our knowledge, there are few, if any, studies describing the clinical course and mortality in septic patients with low severity scores. It is paramount to analyze the features of this specific group of patients since they still carry a certain risk of death and are worth medical attention. Therefore, the aim of the present study was to determine clinical characteristics, trajectories, and causes of death in septic patients with a low predicted mortality risk.

## 2. Experimental Section

### 2.1. Study Setting and Population

This retrospective study was conducted at the National Taiwan University Hospital, a tertiary referral center in Taiwan. Between November 2010 and December 2014, all patients admitted to the medical ICUs with a primary diagnosis of sepsis were eligible for this study. The inclusion criteria were age ≥20 years and an APACHE II score of ≤15 within 24 h of ICU admission. Sepsis was defined as documented or suspected infection determined by retrospective review of the chart, plus organ dysfunction identified as an acute change in the total sequential organ failure assessment (SOFA) score of ≥2 consequent to the infection based on the sepsis-3 definition [2]. The calculation of the SOFA score was retrospectively done by the investigators based on available data from a detailed chart review. Where individual components of the SOFA score were missing, they were assumed to be normal. Overall, the proportions of the missing data were less than 5% for all variables in the analyses. If a patient was admitted to the ICU more than once, only the first ICU admission was counted. The study was conducted in accordance with the Declaration of Helsinki, and the protocol was approved by the Research Ethics Committee of the National Taiwan University Hospital (201507092RINC). The informed consent was waived in view of the retrospective nature of the study and the absence of patient safety concerns.

### 2.2. Data Collection

Data were retrieved via detailed chart review. The following information was recorded: age, sex, comorbidities, indications for ICU admission, primary sites of infection, places of sepsis acquisition (community vs. hospital), causative microorganisms, incident events during the ICU stay (new-onset sepsis and cardiovascular episodes), and lengths of ICU and hospital stays. Comorbidities of interest included chronic kidney disease, chronic lung disease, cardiovascular disease (heart failure and coronary artery disease), diabetes mellitus, liver cirrhosis, malignancy, and stroke [17,18]. Immunocompromised state was considered present if a patient had any of the following: HIV or AIDS, current use of steroids or other immunosuppressive drugs, or any transplanted organ. Sepsis was considered hospital-acquired if the index infection occurred ≥48 h after hospitalization and was deemed community-acquired if the aforementioned criteria were not met [19]. New-onset ICU sepsis was defined as follows: (1) new infection starting more than 48 h after admission to the ICU during the same hospitalization, which met the diagnostic criteria for sepsis described above and required one or more new antibiotic regimens; (2) clinical improvement from the index sepsis lasting for at least 24 h; and (3) a new etiology of infection being identified [20,21,22]. Cardiovascular episodes included any atrial or ventricular arrhythmia causing hemodynamic instability, acute coronary syndrome, myocardial infarction, and ischemic or hemorrhagic stroke. The APACHE II and SOFA scores were calculated based on the worst variables recorded within the 24 h after the patient’s admission to the ICU [23,24].

### 2.3. Outcome Measures

All records of study subjects were extracted until death or hospital discharge. The primary outcome was in-hospital mortality of the study cohort. Also, we sought to determine the clinical trajectories and causes of death in this specific patient population, and to identify factors that would influence their mortality risks. The causes of death in this study were determined by investigators’ consensus, after taking into consideration death certificates and clinical records.

### 2.4. Statistical Analysis

Categorical variables were compared using χ^2^ or Fisher’s exact test, as appropriate, and continuous variables using Student’s t test. Data were presented as number (%), mean ± standard deviation, or median (interquartile range) according to data distribution. Multivariate logistic regression analysis was used to determine independent factors associated with in-hospital mortality, and odds ratios (ORs) with their 95% confidence intervals (CIs) were calculated. All variables with a *p* value of <0.1 in the univariate analysis were entered into the multivariate model. A two-tailed *p* value of <0.05 was considered statistically significant. All analyses were performed using SPSS version 15.0 (SPSS Inc., Chicago, IL, USA).

## 3. Results

### 3.1. Patients

During the study period, 352 (16%) out of 2250 septic patients had an APACHE II score of ≤15 on ICU admission. The mean age of the study cohort was 64.6 ± 18.2 years and 60% of them were male gender. The average APACHE II score on ICU admission was 11.7 ± 2.9 and the majority (83%) had a score of ≥10. Respiratory failure (71%) was the main indication for ICU admission. The most prevalent comorbidities were cardiovascular disease (38%), diabetes mellitus (27%), and malignancy (17%). The lung infection (82%) was the major source of sepsis, and community-acquired sepsis accounted for 53% of the patients. Causative microorganisms were not identified in about half of the study population (Table S1) and Gram-negative bacteria were the most commonly isolated pathogens, accounting for 30% of sepsis episodes.

### 3.2. Clinical Course and Outcome

**Figure 1:**
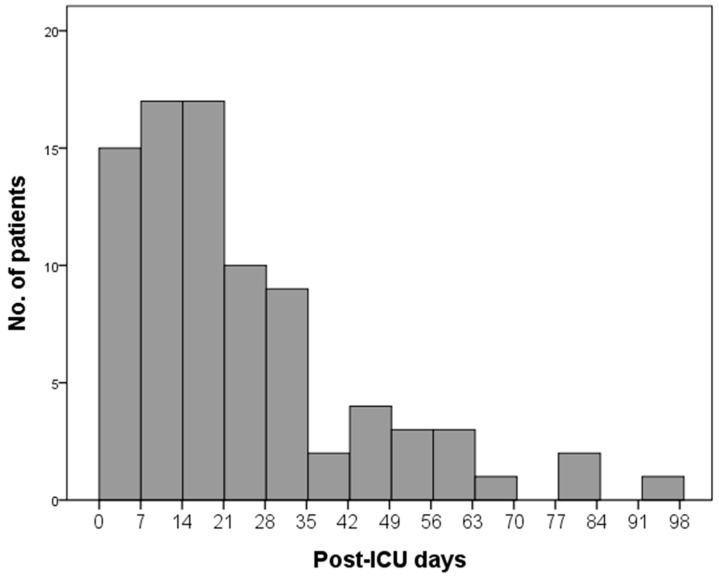
Distribution of deaths according to time post-ICU admission. ICU, intensive care unit.

The median length of the ICU and hospital stays for the study population was 9.5 (6.0–19) and 23 (13–46) days, respectively. In-hospital mortality was observed in 89 (25%) patients (Table 1), and the mortality rate in the first week was 4.3% (15/352), in both second and third weeks it was 4.8% (17/352), and it declined in the subsequent weeks (Figure 1). The majority of the deaths (77/89, 87%) occurred in the first 8 weeks following ICU admission. Univariate comparisons between survivors and non-survivors showed that non-survivors had a higher APACHE II score, more prevalent comorbid malignancy and immunocompromised state, and were more likely to acquire sepsis in the hospital setting. In addition, fungal sepsis was associated with increased mortality, and patients with Gram-positive bacterial sepsis had a favorable outcome (Table S1).

During the ICU stay, new-onset sepsis developed in 99 (28%) patients (Table 2). Cardiovascular events (*n* = 8, 2.3%) were uncommonly observed in the study participants. Of note, new-onset ICU sepsis was associated with a worse outcome in septic patients. Approximately one third (31/89, 35%) of non-survivors succumbed to the index sepsis; however, more patients (34/89, 38%) died of the subsequent sepsis (Table 3).

### 3.3. Predictors of In-Hospital Mortality

Multivariate logistic regression analysis indicated that male gender (OR, 1.828; 95% CI, 1.041–3.209) and comorbid malignancy (OR, 3.274; 95% CI, 1.771–6.052) were independently associated with in-hospital mortality (Table 4). Those patients with hospital-acquired sepsis (OR, 1.728; 95% CI, 1.016–2.938) and a higher APACHE II score (OR, 1.107 per point increase; 95% CI, 1.003–1.223) also had a significantly higher risk of mortality. Moreover, acquisition of new-onset ICU sepsis (OR, 2.342; 95% CI, 1.322–4.149) was an independent risk factor for in-hospital mortality.

## 4. Discussion

Our study elaborated on critically ill septic patients with a low APACHE II score and the main findings were as follows: (a) septic patients with an APACHE II score of ≤15 on ICU admission still carried a significant in-hospital mortality rate of 25% (89/352); (b) a little more than one third (31/89, 35%) of the non-survivors died of the index sepsis; however, even more patients (34/89, 38%) succumbed to the subsequent sepsis; (c) nearly a third (99/352, 28%) of these septic patients developed a new episode of sepsis during their ICU stay; and (d) septic patients with a comorbid malignancy or the index infection acquired in the hospital setting were more likely to experience in-hospital mortality than those without; moreover, new ICU-acquired sepsis represented a poor prognostic sign for the in-hospital outcome. Taken together, the present study encourages more clinical and research efforts directed towards improving care and outcomes of ICU septic patients with a low predicted probability of mortality since 1 in 4 of these low-risk patients cannot survive to hospital discharge during the index admission. Of note, new-onset sepsis in the ICUs was not uncommon and was an independent risk factor for in-hospital mortality among septic patients; thus, strategies to prevent or tackle the development of ICU-acquired sepsis may improve the outcomes for this specific patient population.

To the best of our knowledge, this is the first study to describe the clinical features, course, and outcomes of ICU septic patients with a low risk of death, as predicted by the APACHE II score. The actual in-hospital mortality rate of 25% in our study population with an APACHE II score of ≤15 was higher than that predicted by the original APACHE II model, i.e., an in-hospital mortality rate of 21% in the critically ill with a score of 15 [23]. This observation raises a number of important issues that we ought to discuss. First, there is as yet no ideal and widely-adopted scoring system for septic patients. Most of the scoring models, like the APACHE II score, are derived from general ICU patients and have inconsistent accuracy in predicting mortality in the sepsis population [25]. Undoubtedly, lots of efforts have been exerted to establish a sepsis-specific severity score [26,27,28]; however, none of them have gained widespread acceptance. To date, the APACHE II score remains the most commonly used severity scoring system worldwide for critically ill septic patients. In this regard, the present study suggests that the APACHE II model may underestimate the mortality risk of septic patients in the low score range, and the prognostic information of the APACHE II score in this specific patient population should be interpreted and applied with caution.

Second, in line with prior studies [29,30], we found that hospital-acquired sepsis was significantly associated with higher in-hospital mortality. There are several potential explanations for this finding. Hospital-acquired sepsis may be more likely to be caused by microorganisms with increased virulence and antimicrobial resistance than community-acquired sepsis [31,32]. Also, inappropriate antimicrobial therapy and impaired host defense could play a role in this regard [32,33]. Moreover, recognition of sepsis that develops in the hospital is difficult and this may result in delayed intervention. However, the place of sepsis acquisition (hospital-acquired vs. community-acquired), an identified mortality predictor in our study, is not accounted for in the APACHE II score; thus, this may partly explain why septic patients in the present cohort had a worse in-hospital outcome than predicted by this scoring system. In brief, this study indicates that a simple classification of sepsis into hospital- or community-acquired sepsis would probably improve outcome prediction in ICU septic patients admitted with the diagnosis of sepsis beyond the APACHE II model and is a useful index to have on hand in daily clinical practice.

Third, in the current study, only 15 (17%) of the non-survivors died within the first 7 days of their ICU admission (Figure 1); by contrast, more than 40% of the ICU septic patients succumbed to death within the same time frame in other sepsis studies [34,35]. The discrepancy could be probably attributed to differences in the study populations. We included septic patients with a low severity score at ICU admission in this study, and these patients may have a distinct clinical trajectory and different causes of death as compared to general septic patients in the critically ill setting. To further support this assumption, our study also showed that the majority of the non-survivors died of index sepsis and subsequent sepsis, with the latter accounting for a little more than the former. On the contrary, in a retrospective review of mortality from septic shock at a single center in France, index sepsis was the most common cause of death and subsequent sepsis accounted for a far smaller number of deaths [35]. Moreover, in the large phase III sepsis study Recombinant Human Activated Protein C Worldwide Evaluation in Severe Sepsis [36], more than 80% of the deaths were causally related to the index sepsis [37]. In summary, the above findings emphasize the importance of the present study that specifically described the clinical course and outcome of ICU septic patients with a low APACHE II score. In addition, since subsequent sepsis rather than index sepsis was the leading cause of death among our low-risk septic patient population, this may also justify why a scoring system, measuring severity of illness based on a combination of physiologic variables and patient characteristics on presenting to the ICU, did not exactly forecast their prognosis.

Compelling evidence has indicated sepsis as an immunosuppressive disorder; thus, septic patients who survive the primary insult may still be susceptible to secondary infections [38,39]. In the ICU setting, hospital-acquired infections have been shown to adversely impact on patient outcomes [40,41]. Consistent with previous studies, more than a fourth of our sepsis cohort developed new-onset sepsis throughout the ICU stay and this imposed an additional mortality risk on the study population. Prevention is certainly better than cure and adherence to standard care procedures to prevent hospital-acquired infections is recommended for healthcare providers at all times. In the coming era, the immunostimulatory therapy to restore immunocompetence is a potentially practical way to break the vicious cycle [42]. However, unlike promising results in immuno-oncology [43], immunomodulatory therapy in sepsis remains at its infancy stage, and a lot more work is required to achieve this next major advance in sepsis management [38].

A few limitations to this study need to be pointed out. Our study was conducted in a medical center, where the ICU usually accommodated septic patients with complicated comorbidities not exactly reflected by the APACHE II score. Thus, the observed in-hospital mortality rate in our study cohort may be higher than that obtained in a lower-level care facility. This may limit the generalizability of our study findings; however, as the pioneer study to describe clinical features of these so-called low-risk septic patients, we hope that our report will draw attention to this specific patient population and encourage more studies to investigate this issue across a variety of institutional settings. In addition, given the retrospective design of the present study, reporting and ascertainment bias may affect our study results. For instance, major cardiovascular events were more likely to be documented in the medical chart and were retrieved during data collection; by contrast, minor occurrences would be overlooked in this study. Yet, our analysis did not reveal cardiovascular events to be associated with high mortality risks. Therefore, the minor and perhaps insignificant events may exert little, if any, impact on our study findings and inferences.

## 5. Conclusions

In conclusion, septic patients with a low APACHE II score were at a higher mortality risk than predicted by this scoring system, and subsequent sepsis rather than index sepsis was the primary cause of death in this specific patient population. Further, new-onset sepsis during the ICU stay was not uncommonly encountered in our sepsis cohort and represented a poor prognostic factor. For the first time, this study provides insight into unexpected clinical trajectories and outcomes of septic patients with a low predicted risk of death and highlights the need for researches and clinical attention to this group of patients.

## Figures and Tables

**Table 1:** Characteristics of the study population.

	Survivors	Non-Survivors	
Variables	*n* = 263	*n* = 89	*p* value
Age, years	64.3 ± 18.3	65.5 ± 18.0	0.602
Male gender	151 (57)	60 (67)	0.096
APACHE II ^1^	11.5 ± 3.1	12.5 ± 2.4	0.002
Main reason for admission			
Respiratory failure	187 (71)	64 (72)	0.992
Shock	37 (14)	11 (12)	
Others	39 (15)	14 (16)	
Comorbidities			
Malignancy	31 (12)	30 (34)	<0.001
Liver cirrhosis	5 (1.9)	4 (4.5)	0.238
Cardiovascular disease	97 (37)	35 (39)	0.681
Stroke	6 (2.3)	4 (4.5)	0.280
Chronic kidney disease	21 (8.0)	5 (5.6)	0.461
Chronic lung disease	35 (13)	15 (17)	0.408
Diabetes mellitus	76 (29)	20 (23)	0.239
Immunocompromised state	11 (4.2)	9 (10)	0.037
Place of sepsis acquisition			
Community-acquired	147 (56)	39 (44)	0.049
Hospital-acquired	116 (44)	50 (56)	
Site of infection			
Lung	215 (82)	74 (83)	0.930
Urinary tract	13 (4.9)	3 (3.4)	
Abdomen	18 (6.8)	6 (6.7)	
Others	17 (6.5)	6 (6.7)	

^1^ APACHE, acute physiology and chronic health evaluation.

**Table 2:** Events during the intensive care unit stay.

	Survivors	Non-Survivors	
Events	*n* = 263	*n* = 89	*p* value
New-onset sepsis	62 (24)	37 (42)	0.001
Cardiovascular event	4 (1.5)	4 (4.5)	0.115

**Table 3:** Causes of death for the study population.

	Non-Survivors
Causes of death	*n* = 89
Subsequent sepsis	34 (38)
Index sepsis	31 (35)
Terminal cancer	8 (9.0)
End-stage organ failure	8 (9.0)
Cardiovascular event	3 (3.3)
Others	5 (5.6)

**Table 4:** Multivariate model for factors associated with in-hospital mortality.

Variables	Odds Ratio	95% CI ^2^
Male gender	1.829	1.042–3.209
APACHE II, per point ^1^	1.107	1.003–1.223
Comorbidities		
Malignancy	3.283	1.777–6.065
Immunocompromised state	1.491	0.491–4.525
Hospital-acquired sepsis	1.722	1.014–2.927
Microorganisms		
Gram-positive bacteria	0.148	0.019–1.153
Fungi	2.792	0.760–10.249
ICU events ^3^		
New-onset sepsis	2.405	1.377–4.202

^1^ APACHE, acute physiology and chronic health evaluation; ^2^ CI, confidence interval; ^3^ ICU, intensive care unit.

## Supplementary Materials

The following are available online at https://www.mdpi.com/2077-0383/8/7/1064/s1, Table S1: Causative microorganisms.
