# Supplementary material for: Clinical Trajectories and Causes of Death in Septic Patients with a Low APACHE II Score
Source: J Clin Med. 2019 Jul 20;8(7):1064. doi: 10.3390/jcm8071064 (PMC6678558; doi:10.3390/jcm8071064)
Supplement: Supplementary file 1 [file jcm-08-01064-s001.pdf]

Table S1. Causative microorganisms.

	Full Population	Survivors	Non-Survivors	
Microorganisms	*n* = 352	*n* = 263	*n* = 89	*p* value
Gram-positive bacteria	21 (6.0)	20 (7.6)	1 (1.1)	0.026
Streptococcus pneumoniae	7	6	1	
Staphylococcus aureus	10	10	0	
Others	4	4	0	
Gram-negative bacteria	107 (30)	80 (30)	27 (30)	0.989
Klebsiella pneumoniae	29	23	6	
Pseudomonas aeruginosa	21	15	6	
Acinetobacter baumannii	13	8	5	
Stenotrophomonas maltophilia	10	8	2	
Enterobacter species	6	5	1	
Escherichia coli	10	5	5	
Others	18	16	2	
Fungi	14 (4.0)	6 (2.3)	8 (9.0)	0.010
Pneumocystis jiroveci	12	5	7	
Others	2	1	1	
Virus	12 (3.4)	8 (3.0)	4 (4.5)	0.508
Influenza A virus	7	5	2	
Cytomegalovirus	3	2	1	
Others	2	1	1	
Mycobacteria	8 (2.3)	5 (1.9)	3 (3.4)	0.422
Mycobacterium tuberculosis	6	4	2	
Non-tuberculous mycobacteria	2	1	1	
Multiple microorganisms	18 (5.1)	12 (4.6)	6 (6.7)	0.412
Unidentified	172 (49)	132 (50)	40 (45)	0.392
